# Stillbirth and neonatal mortality in a subsequent pregnancy following stillbirth: a population-based cohort study

**DOI:** 10.1186/s12884-021-04355-7

**Published:** 2022-01-04

**Authors:** Janna W. Nijkamp, Anita C. J. Ravelli, Henk Groen, Jan Jaap H. M. Erwich, Ben Willem J. Mol

**Affiliations:** 1 Department of Obstetrics and Gynecology, University of Groningen, University Medical Center Groningen, Groningen, Netherlands; 2 Department of Medical Informatics, Academic Medical Center Amsterdam, Amsterdam, Netherlands; 3 Department of Epidemiology, University of Groningen, University Medical Center Groningen, Groningen, Netherlands; 4 Department of Obstetrics and Gynecology, Monash University, Monash Medical Center, Clayton, Australia

**Keywords:** Recurrent stillbirth, Neonatal death, Gestational age, Subsequent pregnancy, Perinatal outcome, Obstetric management

## Abstract

**Background:**

A history of stillbirth is a risk factor for recurrent fetal death in a subsequent pregnancy. Reported risks of recurrent fetal death are often not stratified by gestational age. In subsequent pregnancies increased rates of medical interventions are reported without evidence of perinatal benefit. The aim of this study was to estimate gestational-age specific risks of recurrent stillbirth and to evaluate the effect of obstetrical management on perinatal outcome after previous stillbirth.

**Methods:**

A retrospective cohort study in the Netherlands was designed that included 252.827 women with two consecutive singleton pregnancies (1^st^ and 2^nd^ delivery) between 1999 and 2007. Data was obtained from the national Perinatal Registry and analyzed for pregnancy outcomes. Fetal deaths associated with a congenital anomaly were excluded. The primary outcome was the occurrence of stillbirth in the second pregnancy stratified by gestational age. Secondary outcome was the influence of obstetrical management on perinatal outcome in a subsequent pregnancy.

**Results:**

Of 252.827 first pregnancies, 2.058 pregnancies ended in a stillbirth (8.1 per 1000). After adjusting for confounding factors, women with a prior stillbirth have a two-fold higher risk of recurrence (aOR 1.96, 95% CI 1.07–3.60) compared to women with a live birth in their first pregnancy. The highest risk of recurrence occurred in the group of women with a stillbirth in early gestation between 22 and 28 weeks of gestation (a OR 2.25, 95% CI 0.62–8.15), while after 32 weeks the risk decreased. The risk of neonatal death after 34 weeks of gestation is higher in women with a history of stillbirth (aOR 6.48, 95% CI 2.61–16.1) and the risk of neonatal death increases with expectant obstetric management (aOR 10.0, 95% CI 2.43–41.1).

**Conclusions:**

A history of stillbirth remains an important risk for recurrent stillbirth especially in early gestation (22–28 weeks). Women with a previous stillbirth should be counselled for elective induction in the subsequent pregnancy at 37–38 weeks of gestation to decrease the risk of perinatal death.

## Introduction

Stillbirth is a devastating complication of pregnancy. In high-income countries, one in every 200 pregnancies reaching 22 weeks of gestation ends in a stillbirth [1]. Increased maternal age, non-Western ethnic origin, use of alcohol or tobacco and maternal comorbidities as obesity, hypertension, diabetes, preterm birth and small for gestational age fetus (SGA) are risk factors for stillbirth [2]. A history of stillbirth may independently be associated with an increased risk of recurrent stillbirth; previous studies showed an increased risk of fetal death varying from zero to tenfold [3–8].

Cause of stillbirth is complex as there are many contributing and interacting factors varying with gestational age. Risk factors, etiology and the underlying mechanism of the prior stillbirth may influence the risk of recurrent fetal death in a subsequent pregnancy. Reported risks of recurrent fetal death are often not stratified by gestational age, etiology or underlying mechanism of the prior stillbirth. These limitations makes it difficult to counsel parents regarding recurrent risk of stillbirth and to know what level of care to provide in subsequent pregnancies. Previous studies reported increased rates of interventions including higher rates of induction of labor, instrumental deliveries and Caesarean deliveries [6, 9]. However, the perinatal benefit from induction of labor after previous stillbirth is not certain and the best timing for delivery is unsure.

The aim of this study was to assess the recurrence risk of stillbirth and to assess specifically whether stillbirth recurrence depends on the gestational age of stillbirth in the first pregnancy. Furthermore, we investigated the influence of obstetrical interventions on perinatal outcome in pregnancy following stillbirth.

## Methods

We retrospectively evaluated nationwide childbirth data in the Netherlands collected in the Netherlands Perinatal Registry (PRN) from 1999 to 2007, currently named PERINED. The PERINED registry consists of population-based data that contain information on pregnancies, deliveries ≥ 22 weeks of gestation and a birth weight of ≥ 500 g and re-admissions until 28 days after birth. The PERINED database is obtained by a validated linkage of three different registries: the midwifery registry (LVR1), the obstetrics registry (LVR2) and the neonatology registry (LNR) of hospital admissions of new-borns [10–13]. The coverage of the PERINED registry covers approximately 96–98% of all deliveries in the Netherlands. For this study a longitudinal probabilistic linkage dataset is used, which made it possible to analyse the data of the first and second consecutive pregnancies of the same mothers between January 1, 1999 and December 31, 2007. The longitudinal probabilistic linkage procedure is reported in detail elsewhere [10, 11]. In summary, all children from second deliveries who were registered in the PERINED registry were linked to their siblings who were born during the first delivery. The linkage was based on the variables of the birth date of mother, the birth date of a (previous) child and the postal code of mother. The Netherlands Perinatal Registry scientific board gave permission for the analysis of their data to study recurrent stillbirth risk (approval number 11.44). All data in the national registry were anonymous collected by Perined, no further ethical approval was needed under Dutch law and regulations [14].

From our linked population-based cohort, we included all women who delivered from 22 weeks onwards, two subsequent singleton pregnancies (first and second delivery) in the Netherlands between January 1, 1999 and December 31, 2007. We excluded all pregnancies involving reported congenital malformations, all multiple gestations and pregnancies exceeding 43 + 0 weeks of gestation (in the first and/or second pregnancy).

Data of demographic and obstetric characteristics were available from our PERINED registry. Maternal age was categorized into < 25, 25–29, 30–34 and ≥ 35 years. Ethnicity was ascribed by the woman’s healthcare provider. We differentiated between Western Caucasian women (native Dutch women and non-Caucasian women) [15]. The socio-economic status (SES) score is based on mean income level, the percentage of households with a low income, the percentage of inhabitants without a paid job and the percentage of households with, on average, a low education in a 4 digit postal code area [16]. We defined a high, middle and low SES group based on percentile ranges (≤ 25^th^, 25^th^ till 75^th^ or ≥ 75^th^ percentile). Hypertensive disorders included pre-existing hypertension, pregnancy-induced hypertension and (pre)eclampsia. A history of diabetes was defined as pre-existing diabetes or gestational diabetes. Thromboembolic disorders included vein thrombosis, arterial thrombosis and pulmonary embolisms. Placental abruption was a clinical diagnosis eventual supported by placental examination. Preterm birth was defined as < 37 weeks of gestation. Definition of small-for-gestational age (SGA) was a birth weight below the 10^th^ percentile for gestational age based on the Dutch reference curves [17].

The primary outcome assessed was the occurrence of stillbirth in the second pregnancy. Stillbirth was defined as an ante- or intrapartum fetal death ≥ 22 weeks of gestation with a birth weight of ≥ 500 g. Stillbirth rates in both pregnancies were calculated by total number of stillbirths divided by total number of all live births and stillbirths.

First, we stratified our analysis by gestational age periods to establish gestational age-specific stillbirth recurrence rates. Gestational age was based on early ultrasound (usually) or the first day of the last menstrual period. Gestational age periods were categorized as 1) 22 + 0 – 27 + 6 weeks of gestation, 2) 28 + 0 – 31 + 6 weeks of gestation, 3) 32 + 0 – 36 + 6 weeks of gestation and 4) ≥ 37 + 0 weeks of gestation. When calculating gestational age-specific stillbirth recurrence, women with a stillbirth in a given gestational age group in the first pregnancy were the exposed group, while women with a live birth in a given gestational age group in the first pregnancy were the reference group.

Second, we analysed the influence of obstetrical management on perinatal outcome in a subsequent pregnancy. Obstetrical management was categorized as 1) expectant management (defined as no intervention until a spontaneous start of labour) or 2) planned delivery (defined as scheduled start of labour by induction of labour or elective Caesarean section). Perinatal outcomes were defined as stillbirth or neonatal death (death of a new-born within the first 28 days of life). In our supplementary analyses, we restricted the analyses of risk of stillbirth recurrence and neonatal death stratified by obstetrical management a subgroup of births ≥ 34 weeks of gestation, as we hypothesis that in the Netherlands before 34 weeks of gestation no active management will be planned only based on a history of a stillbirth.

Univariate analyses were performed with the Student t test and X^2^ test, as appropriate, to compare baseline characteristics. All statistical tests were 2-sided; a probability value of 0.05 was chosen as the threshold for statistical significance. Logistic regression modelling, which was used to determine gestational age-specific stillbirth recurrence, was expressed as crude odds ratios with 95% confidence interval. In a multivariate analysis, we adjusted for maternal age, low SES, non-Western ethnicity, and a history of male gender, SGA, hypertension, diabetes and placental abruption in the first pregnancy. Cox regression analysis, which was used to analyse stillbirth and neonatal mortality stratified by obstetrical management, was expressed as hazard ratios with 95% confidence interval. Cox regression analyses were in the total population from 22 weeks onwards and in the subpopulation from 34 weeks onwards. We adjusted here for maternal age, low SES, non-Western ethnicity and a history of SGA, hypertension or diabetes in the first pregnancy. Data were analyzed with the SAS statistical software package (version 9.4; SAS Institute Inc, Cary, NC).

## Results

We identified 272.551 women with two consecutive pregnancies in the PERINED database. We excluded 12.155 women with a pregnancy complicated by a congenital anomaly, 7.480 women with multiple gestations and 43 women with a pregnancy > 43 + 0 weeks. Thus, data of 252.827 women with a first and second pregnancy could be studied.

Table 1 shows the results of the baseline characteristics for women with a previous stillbirth (*n* = 2.058) and women with a live birth in the first pregnancy (*n* = 250.769). Women with a stillbirth in their first pregnancy were younger, had a lower social economic status and were more often from a non-Western ethnicity. Hypertensive disorders, preterm birth, SGA and placental abruption were more prevalent among women with a stillbirth in their first pregnancy when compared to women with a live birth.

**Table 1:** Population and clinical characteristics of women with a previous live birth compared to women with a stillbirth in the first pregnancy

**CHARACTERISTICS STUDY POPULATION**								
**BASELINE**	**History of live birth** *n* = 250.769		**History of stillbirth** *n* = 2.058		**Stillbirth in the first pregnancy**			
					Crude OR 95% CI adjusted OR^a^ 95% CI			
Age								
< 25 years	16.951	(6.8)	282	(13.7)	1.42	1.26—1.60	1.12	0.98—1.28
25–29 years	64.067	(25.6)	646	(31.4)	*Ref*		*Ref*	
30–34 years	118.209	(47.1)	753	(36.6)	0.99	0.89—1.10	0.96	0.86 – 1.08
≥ 35 years	51.542	(20.6)	377	(18.3)	1.73	1.49—2.01	1.29	1.10 – 1.52
Low social economic status (< 25^th^ percentile)	51.729	(20.6)	536	(26.0)	1.29	1.17—1.42	1.11	0.99 – 1.25
Non-Western ethnicity	27.375	(10.9)	339	(16.5)	1.55	1.38—1.75	1.21	1.05 – 1.40
Mean interpregnancy interval in months (SD)	30.6	(13.0)	18.3	(9.9)	0.83	0.82—0.83	0.85	0.84 – 0.87
**GENERAL HISTORY**								
Diabetes	1.749	(0.7)	21	(1.0)	1.47	0.95—2.26	1.72	1.08 – 2.76
Hypertensive disorders	31.275	(12.5)	370	(18.0)	1.54	1.37—1.72	0.89	0.78 – 1.02
Thromboembolic disorders	527	(0.2)	6	(0.3)	1.39	0.62—3.11	0.98	0.39 – 2.45
**OBSTETRIC OUTCOME (first pregnancy)**								
Gender first born: male	128.435	(51.2)	1.128	(54.8)	1.16	1.06—1.26	1.02	0.93 – 1.13
Small for gestational age (< 10^th^ percentile)	37.004	(14.8)	1.228	(59.7)	8.55	7.82—9.34	4.66	4.19 – 5.18
Placental abruption	331	(0.1)	124	(6.0)	48.53	39.3—59.9	13.7	10.3 – 18.2

Results are given as n (%) unless otherwise specified. *OR* odds ratio, *CI* confidence interval, *Ref* Reference

^a^Adjustment for baseline characteristics (maternal age, low SES, non-Western ethnicity), general history (history of diabetes, pre-existing hypertension and thromboembolic disorders) and obstetric outcome in first pregnancy (male gender, SGA, pregnancy-induced hypertension and pre-eclampsia)

In the subsequent pregnancy overall stillbirth rates were lower for both women with a previous live birth as well as women with a stillbirth, but women with a previous stillbirth have a significant higher risk of recurrence (5.8 per 1000 versus 3.2 per 1000; adjusted OR 1.96, 95% CI 1.07–3.60). When we stratified stillbirth rates by gestational age in the first pregnancy, women with a first stillbirth at early gestation (22 + 0 until 27 + 6 weeks), had the highest risk of recurrent stillbirth (15.0 per 1000 versus 8.4 per 1000; adjusted OR 2.25, 95% CI 0.62–8.15). The risk of recurrent stillbirth decreases when previous stillbirth occurred after 28 + 0 weeks of gestation, and after 37 weeks of gestation the risk was even lower than for women with a previous live birth (1.1 per 1000 versus 3.0 per 1000; adjusted OR 0.35, 95% CI 0.05–2.50). (Table 2).

**Table 2:** Crude and adjusted odds ratios of total and gestational-age specific recurrent risk of stillbirth in women with a previous stillbirth compared to women with a previous live birth

Gestational age	History of live birth			History of stillbirth			Recurrent stillbirth Crude OR 95% CI Adjusted OR^a^ 95% CI			
	total	n stillbirths	Stillbirth rate	total	n stillbirths	Stillbirth rate				
22 + 0 – 27 + 6 weeks	598	5	8.4	401	6	15.0	1.80	0.55—5.94	2.25	0.62—8.15
28 + 0 – 31 + 6 weeks	3.697	31	8.4	480	4	8.3	0.99	0.35—2.83	2.43	0.62—9.54
32 + 0 – 36 + 6 weeks	13.134	57	4.3	272	1	3.7	0.85	0.12—6.14	0.61	0.08—4.86
≥ 37 + 0 weeks	233.340	710	3.0	905	1	1.1	0.36	0.05—2.58	0.35	0.05—2.50
Total	250.769	803	3.2	2.058	12	5.8	1.83	1.03—3.23	1.96	1.07—3.60

Results are given as risk of stillbirths per 1.000 births. *OR* odds ratio, *CI* confidence interval

^a^Adjustment for maternal age, low SES, non-Western ethnicity, and a history of male gender, SGA, hypertension, diabetes and placental abruption in the first pregnancy

Women with a history of stillbirth had more often a planned delivery (73.8% versus 28.9%). If the birth started by induction of labor, the rate of obstetrical interventions was comparable to women with previous live birth (instrumental delivery 8.2% versus 7.0%; emergency Caesarean Sect. 8.4% versus 8.4%). (Table 3).

**Table 3:** Obstetrical management in the second pregnancy of women with a previous stillbirth compared to women with a live birth

Obstetrical management in the second pregnancy	History of live birth *n* = 250.769		History of stillbirth *n* = 2.058		*p* value
**Planned delivery**	72.459	(28.9)	1.518	(73.8)	< 0.001
Elective C-section	17.425	(24.0)	309	(20.4)	
Induced and spontaneous delivery	43.897	(60.6)	957	(63.0)	
Induced and instrumental delivery	5.059	(7.0)	124	(8.2)	
Induced and emergency caesarean section	6.078	(8.4)	128	(8.4)	
**Expectant management**	178.310	(71.1)	540	(26.2)	< 0.001
Spontaneous in labor and spontaneous delivery	165.158	(92.6)	439	(81.3)	
Spontaneous in labor and instrumental delivery	5.802	(3.3)	52	(9.6)	
Spontaneous in labor and emergency caesarean section	7.350	(4.1)	49	(9.1)	

We analyzed the effect of obstetrical management on the recurrence of stillbirth and the risk of neonatal death in a subsequent pregnancy after stillbirth. The recurrence of stillbirth compared to women with a previous live birth, was higher in the group of women with expectant management (expectant management 9.3 per 1000 versus 1.5 per 1000; adjusted OR 5.64, 95% CI 2.31–13.8). (Table 4). In our sub analysis (all pregnancies of ≥ 34 weeks of gestation), the recurrence of stillbirth is still higher in the expectant management group, but this is no longer significant (1.9 per 1000 versus 0.9 per 1000; adjusted OR 2.21, 95% CI 0.31–15.8). (Table 4).

**Table 4:** Influence of obstetrical management on stillbirth recurrence in a subsequent pregnancy after stillbirth

	**History of live birth**			**History of stillbirth**			**Recurrent stillbirth in second pregnancy**			
	total	n stillbirths	Stillbirth rate	total	n stillbirths	Stillbirth rate	Crude HR	95% CI	Adjusted HR ^a^	95% CI
**GA ≥ 22 + 0—< 43 + 0 weeks**	250.769	803	3.2	2.058	12	5.8	2.36	1.33–4.17	1.95	1.10–3.46
Expectant management	178.310	274	1.5	540	5	9.3	7.64	3.15–18.5	5.64	2.31–13.8
Planned delivery	72.459	529	7.3	1.518	7	4.6	0.81	0.38–1.70	0.74	0.35–1.56
**GA ≥ 34 + 0—< 43 + 0 weeks**	248.547	474	1.9	1.996	2	1.0	0.83	0.21–3.35	0.71	1.18–2.84
Expectant management	177.024	167	0.9	519	1	1.9	3.01	0.42–21.5	2.21	0.31–15.8
Planned delivery	71.523	307	4.3	1.477	1	0.7	0.25	0.04–1.77	0.24	0.03–1.70

Results are given as risk of stillbirth per 1.000 births. *GA* gestational age, *HR* hazard ratio, *CI* confidence interval

^a^Adjustment for maternal age, low SES, non-Western ethnicity and SGA

The risk of neonatal death is higher in the group of women with a history of stillbirth (all pregnancies of ≥ 22 + 0 and < 43 + 0 weeks of gestation) compared to women with a previous live birth, both in expectant management group (5.6 per 1000 versus 1.3 per 1000, adjusted OR 5.30, 95% CI 1.69–16.6) and in the group of women with a planned delivery (5.3 per 1000 versus 1.8 per 1000, adjusted OR 1.96, 95% CI 1.15–3.34). The risk of neonatal death after ≥ 34 + 0 weeks of gestation overall decreased. However, the risk remained higher for women with a previous stillbirth, especially in the group with an expectant management (3.9 per 1000 versus 0.6 per 1000, adjusted OR 10.0, 95% CI 2.43–41.1). A planned delivery for women with a previous stillbirth, decreases the risk of neonatal death in this specific group (2.0 per 1000 versus 1.0 per 1000, adjusted OR 4.02, 95% CI 1.22–13.2). (Table 5).

**Table 5:** Influence of obstetrical management on neonatal outcome in a subsequent pregnancy after stillbirth

	**History of live birth**			**History of stillbirth**			**Neonatal mortality in second pregnancy**			
	total	n neonatal deaths	Neonatal death rate	total	n neonatal deaths	Neonatal death rate	Crude HR	95% CI	Adjusted HR ^a^	95% CI
**GA ≥ 22 + 0—< 43 + 0 weeks**	250.769	353	1.4	2.058	11	5.3	5.14	2.82–9.38	4.62	2.51–8.49
Expectant management	178.310	225	1.3	540	3	5.6	5.22	1.77–17.3	5.30	1.69–16.6
Planned delivery	72.459	128	1.8	1.518	8	5.3	4.24	2.07–8.71	1.96	1.15–3.34
**GA ≥ 34 + 0—< 43 + 0 weeks**	248.547	171	0.7	1.996	5	2.5	7.65	3.13–18.7	6.48	2.61–16.1
Expectant management	177.024	102	0.6	519	2	3.9	11.5	2.84–46.8	10.0	2.43–41.1
Planned delivery	71.523	69	1.0	1.477	3	2.0	4.59	1.42–14.8	4.02	1.22–13.2

Results are given as risk of neonatal death per 1.000 births. *GA* gestational age, *OR* odds ratio, *CI* confidence interval

^a^Adjustment for maternal age, low SES, non-Western ethnicity and SGA

## Discussion

We found that women with a prior stillbirth have a two-fold higher risk of recurrent stillbirth compared to women with a live birth in their first pregnancy. Risk of recurrence is highest with a stillbirth between 22 and 28 weeks of gestation in the first pregnancy. The recurrence risk decreases after a stillbirth from 32 weeks onwards in the first pregnancy.

Recurrent stillbirth is higher after expectant management in the second pregnancy. Risk of neonatal death is higher in women with a history of stillbirth and this risk increases with expectant obstetric management, especially in the population of 34 weeks and higher.

In our cohort, women with a stillbirth in their first pregnancy have a higher incidence of known risk factors for fetal death as lower social economic status, non-Western ethnicity and maternal diseases as hypertensive disorders and diabetes [18, 19]. In addition, the first pregnancy of these groups is more often complicated by placental abruption. After adjustment for these potential contributory factors for fetal death in a subsequent pregnancy, we found that women with a prior stillbirth have a significant two-fold higher risk of recurrence in their subsequent pregnancy. Analysis of stillbirth recurrence stratified by gestational age in the first pregnancy showed a specific pattern. The highest risk of recurrence occurs after a previous stillbirth at early gestation (< 28 weeks). If the previous stillbirth occurred after 32 weeks of gestation, the risk of is the same compared to women with a prior live birth. In women with a previous term stillbirth (≥ 37 + 0 weeks), the recurrent risk of stillbirth even disappears.

The found two-fold recurrent risk of stillbirth for the whole population, is in accordance with other publications. Bhattacharya et al. analyzed a cohort of 309.304 women in Scotland between 1981 and 2000, and established a recurrent risk of stillbirth of OR 1.94 (99% CI 1.29–2.92) [3].

Our gestational-age specific recurrent stillbirth rates are not consistent with previous publications on gestational-age specific recurrence of stillbirth. Sharma et al. analyzed 261.384 relatively low-risk women (maternal age < 35 year and non-smoking) using data from the Missouri maternally linked database from 1978 till 1997. Recurrent risks after prior stillbirth were significantly increased in every gestational age period, with adjusted hazard ratios of 10.3 (95% CI 6.1–17.2) in early gestation (20–28 weeks) and 2.5 (95% CI 1.0–6.0) after 28 weeks of gestation [5]. Another large population-based cohort study was performed based on the Medical Birth Registry of Norway in the period between 1967 till 2004 where in total 567.148 women with two consecutive pregnancies were studied. During the 38-year of observation they found very high relative odds of 27.9 (95% CI 21.9–35.6) for early gestation (20–27 weeks) and 4.2 (95% CI 3.2–5.5) in mid/late gestation (≥ 28 weeks) [20]. One important difference between our study and previous studies on gestational-age specific recurrence of stillbirth, is the fact that potential contributing risk factors were taken into account in our analyses. Previous studies on gestational-age specific recurrence did not adjust their stillbirth rates for maternal diseases or pregnancy complications in the first pregnancy like placental abruption, preterm birth or SGA, while underlying maternal pathology may affect the subsequent pregnancy [5, 20]. In addition, there are differences between the study populations and studied time-period which may partly explain the differences between previous studies and our results.

We found in our study that women with a history of stillbirth have more obstetrical interventions in the second delivery, which is comparable with previous studies [6, 21, 22]. We also analyzed the effect of obstetrical management on perinatal outcome. In the total group (all pregnancies of ≥ 22 + 0 and < 43 + 0 weeks of gestation), recurrent stillbirth risk is higher with expectant management. After 34 weeks of gestation the risk is no longer significant influenced by obstetrical management, although most obstetricians choose for a planned delivery in women with a history of stillbirth (73.8%). In our cohort, women with a previous stillbirth have more than five-fold higher risk of neonatal death in the consecutive pregnancy compared to women with a previous live birth. We found a great effect of obstetrical management on neonatal death; after 34 weeks of gestation the risk of neonatal death increases to a tenfold risk in the expectant management group and decreases to fourfold risk with planned delivery. Salihu et al. [23]reported an elevated risk for neonatal mortality after previous stillbirth, with adjusted hazard ratio of 3.23 (95% CI 1.12–4.94) [23]. Getahun et al [24] found even a higher risk of neonatal mortality in a subsequent pregnancy (aOR 7.1, 95%CI 3.2–15.7). They suggest a shared etiology in both pregnancies including vasculopathy, uteroplacental under perfusion, chronic hypoxia and placental ischemia [24]. This is supported by other studies; Monari et al [25] showed an increased risk of adverse neonatal outcome for women with a previous stillbirth related to placental vascular disorders compared to unexplained stillbirth or other causes of stillbirth (aOR 2.1, 95% CI 1.2–3.8) [25]. Ofir et al. [26] found in subsequent pregnancy following placental stillbirth a 10 times higher risk of IUGR [26]. Wood et al. [27] reported a higher risk of subsequent antepartum stillbirth for women with a previous stillbirth (RR 4.28, 95% CI 2.76–6.65), but especially for women whose first stillbirth was small for gestational age (RR 10.39, 95% CI 5.81–18.59) [27]. They believe that these women may benefit from active obstetrical management including enhanced surveillance.

To our best knowledge, this is the first study which presented data on recurrent stillbirth and risk of neonatal death stratified by obstetrical management after previous stillbirth and shows that after 34 weeks of gestation there is a greater benefit on neonatal outcome with active obstetrical management. Studies which focus on outcome after preterm delivery supports our findings on neonatal death. Lisonkova et al. [28] reported that in countries with higher rates of preterm birth (> 32 weeks of gestation), neonatal death rates are lower. The authors suggest that this inverse association is a result of an increase in medically indicated late preterm deliveries to prevent perinatal death [28]. With our data, we are not able to identify a common pathophysiological pathway to explain our findings since we have no reliable information regarding causes of stillbirth and neonatal death.

### Clinical implications

First, we have established that a history of a stillbirth remains an important risk for recurrent stillbirth, but the gestational-age specific risk is no longer present for each gestational-age group, which will allow better counselling.

Second, we believe that based on our data, we provide enough evidence to support the current opinion that women with a previous stillbirth should be offered elective induction in the subsequent pregnancy at 38–39 weeks of gestation to decrease the risk of neonatal death [21]. Our findings showed also that active management does not result in a higher rate of instrumental delivery or emergency Caesarean section.

### Research implications

We found some important new directions for further research: we should focus on determining underlying pathophysiological mechanism of early stillbirth (between 22 and 28 weeks of gestation) and the continuing biological pathway between 2 pregnancies resulting in neonatal death in the subsequent pregnancy. In addition, we should look for improvements in obstetrical care like better surveillance or possible interventions for women with a stillbirth in the first pregnancy.

### Strengths

The main strength of this study is the size and composition of the cohort. The coverage of the PERINED registry is approximately 96–98% of all births in the Netherlands. The registration of obstetricians is nearly complete (> 99%), and only circa 1–2% general practitioners and 2–3% midwifes are non-reporting. It is unlikely that we missed some cases of recurrent stillbirth, since a stillbirth is an indication of referral to an obstetrician.

### Limitations

We acknowledge some limitations and possible weaknesses in our study. First, we were not able to adjust the effect of maternal body mass index (BMI) and smoking during pregnancy. Maternal weight and length are not registered in the national perinatal registry. Smoking during pregnancy is an item in the registry, but this information was not used because of the low prevalence (0.4%) due to underreporting. In general, about 10–15% of pregnant women smoke during their pregnancy [29]. Second, there are some limitations of longitudinal linkage which are already described elsewhere. However, additional analysis after the longitudinal linkage procedure showed that the longitudinal liked dataset was comparable to the national pregnancy characteristics in the Netherlands [11]. Therefore it is well applicable for analysis of perinatal outcome. Finally, we have no data available on the diagnostic work-up after the stillbirth and the identified cause of fetal and neonatal death. We are not able to investigated potential underlying pathophysiological mechanisms of recurrence of stillbirth or neonatal death after previous stillbirth.

## Conclusions

Women with a prior stillbirth have a two-fold higher risk of recurrence compared to women with a live birth in their first pregnancy. Risk of recurrence is highest between 22 and 28 weeks of gestation, and decreases after 32 weeks. Risk of neonatal death after 34 weeks of gestation is higher in women with a history of stillbirth. Therefore they should be counselled for elective induction in the subsequent pregnancy at 37–38 weeks of gestation to decrease the risk of neonatal death.

## Data Availability

The data that support the findings of this study are available, upon reasonable request, from The Netherlands Perinatal Registry (www.perined.nl) and the authors. There are restrictions apply to the availability of these national registry data, which were used under license for the current study, and so are not publicly available.

## Abbreviations

SGA: Small for gestational age
PERINED/PRN: Netherlands Perinatal Registry
SES: Social economic status
BMI: Body mass index
